# No longer hype, not yet mainstream? Recalibrating city digital twins' expectations and reality: a case study perspective

**DOI:** 10.3389/fdata.2023.1236397

**Published:** 2023-11-02

**Authors:** Stefano Calzati

**Affiliations:** Department of Urbanism, Section Urban Data Science, Delft University of Technology, Delft, Netherlands

**Keywords:** city digital twin, sociotechnical approach, data governance, urban development, European Union

## Abstract

While the concept of digital twin has already consolidated in industry, its spinoff in the urban environment—in the form of a City Digital Twin (CDT)—is more recent. A CDT is a dynamic digital model of the physical city whereby the physical and the digital are integrated in both directions, thus mutually affecting each other in real time. Replicating the path of smart cities, literature remarks that agendas and discourses around CDTs remain (1) tech-centered, that is, focused on overcoming technical limitations and lacking a proper sociotechnical contextualization of digital twin technologies; (2) practice-first, entailing hands-on applications without a long-term strategic governance for the management of these same technologies. Building on that, the goal of this article is to move beyond high-level conceptualizations of CDT to (a) get a cognizant understanding of what a CDT can do, how, and for whom; (b) map the current state of development and implementation of CDTs in Europe. This will be done by looking at three case studies—Dublin, Helsinki, and Rotterdam—often considered as successful examples of CDTs in Europe. Through exiting literature and official documents, as well as by relying on primary interviews with tech experts and local officials, the article explores the maturity of these CDTs, along the Gartner's hype-mainstream curve of technological innovations. Findings show that, while all three municipalities have long-term plans to deliver an integrated, cyber-physical real-time modeling of the city, currently their CDTs are still at an early stage of development. The focus remains on technical barriers—e.g., integration of different data sources—overlooking the societal dimension, such as the systematic involvement of citizens. As for the governance, all cases embrace a multistakeholder approach; yet CDTs are still not used for policymaking and it remains to see how the power across stakeholders will be distributed in terms of access to, control of, and decisions about CDTs.

## 1. Introduction

In the last few years, the concept of Digital Twin (DT) has gained traction both in the industry and the public sector, as well as in academia. The term DT was first coined by Grieves (2002) to mean a virtual/digital representation of a physical object, such as an engineering component or system. In order to have a proper DT, the existing physical object and its digital simulation must be fully integrated in both directions, so that “a change made to the physical object automatically leads to a change in the digital object and vice versa” (Fuller et al., 2020). From the industrial sector, the term soon migrated to cover digital duplications of business operations, institutions, labor forces, arriving to be extended to one's own persona (“personal digital twin,” de Kerckhove, 2021) and the urban environment (“city digital twin,” Boje et al., 2020; Nochta et al., 2021; Papyshev and Yarime, 2021; Shahat et al., 2021; Wan et al., 2023). Notably, a City Digital Twin (CDT) is a 3D *dynamic model* that can help *synthesize data* from various sources (e.g., geospatial information systems, archival data, Internet of Things) to create an integrated *real-time knowledge* of the city. As such, CDTs bear high expectations from tech experts, city officials, and policymakers to tackle the complex problems affecting cities. However, replicating smart city agendas and discourses, literature (Nochta et al., 2021; Papyshev and Yarime, 2021) remarks that CDTs currently remain (1) tech-centered, that is, focused on overcoming technical limitations, while overlooking a proper social contextualization of the development and use of these technologies and (2) practice-first, entailing a hands-on approach which often lacks a long-term strategy and a comprehensive governance for the management of CDT projects. Moving beyond high-level abstract conceptualizations, it remains unclear what a CDT can do, how, and for whom.

Gartner (2019) identified DT as one of the top 10 strategic technological trends. Two years earlier, the Gartner hype cycle positioned DT half-way to the peak of the curve, expecting to reach mainstream adoption within 10 years (cf. Forbes, 2017). From today's standpoint (September 2023), according to 2017's Gartner hype cycle, DT should have overcome the peak of the hype, entering a descent marked with disillusionment but also practical implementations. From this standpoint, the article explores the current state of implementation as well as prospects and challenges of CDTs in three leading European cities—Dublin, Helsinki, and Rotterdam—contributing to a cognizant study of these projects along Gartner's hype-mainstream curve.

Tackling the limits identified above of current CDTs, the assessment of the maturity of the chosen CDTs will be conducted along three axes: (a) technical, (b) sociotechnical, and (c) governance. This, in turn, will allow to have a better understanding of the current functionalities of CDTs and their use in context. To do so, the analysis will triangulate existing literature on CDTs and the digitalization of the urban environment, with official documents related to the identify case studies, and the voice of tech experts and municipality officials directly involved in these initiatives.

The article is divided as follows: in Section 2 the Gartner's hype cycle is described as a reference to the prosecution of the argument; in Section 3 the concept of (city) digital twin is surveyed and inscribed into the broader research stream of smart cities. Section 4 identifies the main limits of current (CDT) projects; Section 5 unpacks the research questions of the article and outlines the methodology followed; Section 6 provides an overview of the three European CDTs analyzed—Dublin, Helsinki, Rotterdam—triangulating existing literature on sociotechnical and governance approaches to the digitalization in/of the urban environment, gray literature about the case studies, and purposefully collected interviews with relevant actors at the lead of these projects; Section 7 discusses the major findings across the case studies, assessing their CDTs' maturity and what these are used for. Section 8 concludes the article, identifying limitation of the present study and possible future lines of research.

## 2. (City) digital twin between hype and mainstream

The Gartner hype cycle is a model describing the five main stages through which emerging technologies typically go in their evolution and societal acceptance. The first stage is the “innovation trigger,” that is, when a concept or prototype is introduced within a small expert niche; the second stage is the “peak of inflated expectations,” characterized by shared (over)positive hype surrounding the potentials of the new technology; the third stage is the “trough of disillusionment,” marked by a deflation of the hype and the identification of major challenges to the technology's development and impact; the fourth stage is the “slope of enlightenment,” during which a realistic understanding of the technology is achieved and concrete applications emerge; the last stage is the “plateau of productivity,” when the technology goes mainstream, being developed and used by an increasingly wider array of actors.

In Forbes (2017), the Gartner hype cycle positioned DT half-way to the peak of hype cycle model, expecting to reach mainstream adoption within 10 years. From today's standpoint (September 2023), according to 2017's Gartner hype cycle, DT should have overcome the peak of the hype and entered the descent phase marked by disillusionment as well as practical implementations. This understanding, however, demands contextualization: as van Lente (1993) notes, the path an emerging technology takes is part and parcel of a complex interplay of actors, expectations, and types of innovation. In this respect, Gartner's hype cycle is intended here as descriptive of the evolution of CDTs and not prescriptive (on the limits of this latter view, cf. Dedehayir and Steinert, 2016). To move from hype toward mainstream inevitably implies that big expectations about CDTs are shrunk into operational projects, especially when the new technology enters an already dynamic environment populated by multiple actors (Van Lente et al., 2013), such as the case with smart cities. From this standpoint, the article explores the prospects and challenges of current CDTs contributing to a cognizant understanding along the hype-mainstream curve.

## 3. Revamping the smart city: from digital twin to city digital twin

Initially, engineers used the term DT to designate the digital replication of complex and costly motors and installations such as turbines and rotors, the idea being to facilitate their real-time monitoring and management. A machine's digital twin might include not only ongoing reporting on its functioning, but also the history of its maintenance, that of the occasional break-down, as well as the source and coordinates of all parts' suppliers and records. In many cases, the digital twin enables automatic repairs just as it regulates normal functions, and it allows simulating the impact of possible defects and ameliorations, providing a viable, cost efficient and safe testing tool. Fuller et al. (2020) reviewed existing literature on DT to reach a consensual definition: according to their study, the trading mark of a DT is that an existing physical object or process and its digital counterpart are integrated in *both directions*, thus *mutually* affecting each other in real time. This means that, as a model, a digital twin gets repeatedly updated through real-time data coming from and about the physical object or process it represents; in turn, the digital version can, at any time, affects or steers the behavior of its physical counterpart.

This understanding becomes even more radical as soon as the concept of DT is applied to contexts other than the industry and the engineering sector. As Batty (2018) writes “since its inception, the concept has broadened and loosened somewhat in that it is now used to characterize a variety of digital simulation models that pertain to social and economic systems as well as physical systems.” This is the case, for instance, with cities, which are, at once, a locus of digital innovation par excellence (Jacobs, 1969) and a major target of that same innovation through smart city agendas. In fact, merging these agendas with the concept of DT, currently City Digital Twins (CDT) are being developed across Europe, such as in Dublin (Dublin City Council, 2022), Helsinki (City of Helsinki, 2022), Rotterdam (Municipality of Rotterdam, 2023), among others. A CDT can help synthesize data from various sources (e.g., GIS, archival data, social media) to create an integrated knowledge of the city, as well as scenario simulations, both in the short term and long term. According to Shahat et al. (2021), CDT's applications can be broadly divided into five themes: data management, visualization, situational awareness, planning and prediction, and integration and collaboration. As such, a CDT bears high expectations from tech experts, city officials, policymakers as well as private companies, constituting a tech-driven basis to tackle the complex problems affecting cities, from mobility, to energy consumption to logistics. As Papyshev and Yarime (2021) write, “city digital twins (CDTs) create opportunities for city officials to embrace the notion of simulation governance and expand the reach of contemporary planning techniques.” Along this line, Boje et al. (2020) add that the concept of CDT “conveys a holistic socio-technical and process-oriented characterization, by leveraging the synchronicity of the cyberphysical bi-directional data flows.” More than a mere replica of the object or process lifecycle, then, the CDT expands to achieve a life of its own that fosters a unique sociotechnical dimension—and this demands constant monitoring, regulation, and control. In fact, the encounter between DT technologies and the urban environment is nothing particularly unexpected; it represents the latest instantiation of smartening approaches to the city. However, moving beyond high-level conceptualizations, at present it is still unclear what a CDT can do, how, and for whom, requiring a case study-based exploration beyond high-level conceptualizations.

## 4. Issues of city modeling and urban development

As Mattern (2021) notes, far from being systems that can be approached as machines, that is, as something to be broken into parts and then processed and recombined, cities are “hybrid complex systems” (Portugali, 2011) composed of biotic and artificial elements, whose mingling creates a whole unique dimension. This means that cities cannot be studied by isolating either their elements or interactions, but require rather to be studied as a whole, insofar as they manifest emergent behaviors that are very difficult to predict. On this point, Bettencourt (2015) contends that “the challenge for a modern science of cities is to define urban issues in their own right and to seek integrated solutions that play to the natural dynamics of cites.” The idea of resorting to integrated solutions characterizes the need to merge—since the outset—the digital and the physical into orchestrated strategies for the conception, design, implementation, and use of technologies *for* the city (rather than simply *in* the city).

When digital twin technologies are implemented in the urban environment, intertwined issues of modeling design and governance emerge (Kitchin et al., 2021). Far from constituting a mirror of the city, a CDT delivers a *representation* whose heuristics depends on tech affordances—*what* the digital twin as a tech-based model *can* grasp—and non-technical aspects that have to do with *how* to design and *use* (by whom and for which purposes) such a real-time model. As Shahat et al. (2021) note, to develop a CDT entails the participation of three actors—government, industry, and citizens—and demands “continuous coalition toward longer term objectives such as sustainability, resilience, and sustaining growth.” It is therefore necessary to conceive and design CDTs as part of a *multistakeholder sociotechnical* process, by which it is informed and which, in turn, it contributes to inform. In this respect, Nochta et al. (2021) point out that “the usefulness of CDTs in decision-making depends on the success of reframing high-level policy goals into practical policy problems to which the model can suggest solution options. This reframing exercise must be informed by in-depth local knowledge and preferences and thus requires a participatory approach.” In a similar vein, speaking of smart cities Cardullo and Kitchin (2019) call for “more extensive public consultation, collaboration and co-production” when it comes to the smartening of cities through digital technologies. In fact, tech implementation in/for the urban environment is never a neutral affair: how, where, and which technology is deployed are socio-economically loaded questions. Currently, the nexus between tech modeling and implementation, on the one hand, and urban planning and development, on the other hand, is not systematically addressed.

According to recent literature (Nochta et al., 2021; Papyshev and Yarime, 2021; Shahat et al., 2021), agendas and discourses around CDTs tend to follow up on earlier smart city applications, adopting a techno-centric practice-first approach. This means that these projects (1) focus on how to overcome technical limitations (e.g., data interoperability, data semantics, data fusion), without exploring the societal dimension of digital twin technologies; (2) largely lack a governance framework to strategically orient and systematize the use of digital twin technologies for the city.

On the one hand, a tech-centered approach often represents an ally to both technological investments and the delivery of hands-on economic-efficient fixies to complex urban problems. Yet an over-focus on the technical side, often led by tech companies keen to sell their products to as many cities as possible (Kummitha, 2020), risks translating into an enduring gap between the technical and the social (Kalpokas, 2022). Following up on this, Kitchin et al. (2021) warn about the risk for CDTs to “decontextualize a city from its history (…) and the everyday experiences of people living in the city,” with the consequence of overlooking socio-economic and environmental shortcomings of smart solutions, in favor of an understanding of technology as an all-disrupting *and* all-fixing driver: “inequality and poverty,” Viitanen and Kingston (2014) contend, “do not often feature in smart city debates, but the technological fixes in smart cities will have distributional consequences under which there are winners and losers.” To avoid reducing the digital twinning of the urban environment to a neoliberal product (Dembski et al., 2020), it is necessary to regard it as a process that is as much techno-economical as societal (Caprari et al., 2022; Calzati and van Loenen, 2023).

On the other hand, a practice-first approach is often disjoined from a long-term vision. To improve emerging technologies by testing them directly on real-life scenarios is a consolidated practice for advancing technological innovation in/of the city. And yet, this should not exempt tech developers and city officials from inscribing new technologies into a broader frame. A practice-first approach that concentrates too much on “having things done” turns technology into an end, rather than a mean, avoiding an analysis of long-term enablers to digitalization. For instance, Papyshev and Yarime (2021) claim that, while a CDT *might* prove effective for arriving at informed decisions, “the challenges of utilizing CDTs in the process of policymaking from a less engineering-oriented perspective are rarely discussed.” Similarly, Nochta et al. (2021) note that the development and implementation of CDT projects tend to “overlook the necessity and costs of individual (upskilling) and organizational (collaboration) learning,” thus lacking a cognizant discussion on the contextual factors facilitating the city-oriented digitalization of the urban environment. More broadly, a practice-first approach misses to establish an ecosystem whereby urban policymakers, tech innovators, the public sector, private actors, academia, and citizens can define and pursue orchestrated strategies of action (Cazacu et al., 2020).

Overall, these trends call for research that moves beyond the fixing of CDTs' technical limitations and rather explores (a) a sociotechnical approach to CDTs and (b) a long-term strategic governance for CDTs.

## 5. Assessing city digital twins: research question and methodology

While the concept of CDT is increasingly adopted to describe something more than the “traditional” smartening of the city, concretely what a CDT is meant to do, how, and especially for whom are still gray areas that beg for investigation. To do so, the article will explore three CDT projects in Europe—Dublin, Helsinki, and Rotterdam—which are often regarded as leading examples in the digital twinning of the urban environment.

These case studies have been chosen because they are among the earliest, currently most consolidated, and still ongoing CDT projects in Europe. Notably, the “Smart Dublin” initiative (Dublin City Council, 2022) dates back to mid 2010s similarly to Rotterdam's “Digitale Stad” (trans. “Digital City”) which has its roots in the first half of 2010s; as for Helsinki, the municipality started to develop a city-wide digital twin in the early 2000s. To this it must be added that these three cities share similar population size (all three around 500.000 inhabitants), as well as similar multi-disciplinary objectives as far as the development of CDTs is concerned (e.g., applied to energy, mobility, logistics, safety, etc.). This means that, while the implementation of digital twin models might (initially) occur at specific spatial scales (e.g., neighborhood or districts) and/or related to a pilot sector (e.g., energy or mobility), the strategy by these municipalities maintains a city outlook, even when the plan is to develop more interconnected CDTs.

On the one hand, the review of the case studies is informed by primary literature (academic articles) and gray literature (e.g., reports, official websites) in order both to understand how CDTs are conceived and discursively framed, as well as to highlight strengths and weaknesses based on exiting literature. For Dublin, the website “Smart Dublin” (Dublin City Council, 2022) was consulted, as well as a public interview to the head of the initiative (Bee Smart City, 2021); for Rotterdam, the website “Digitale Stad” (Municipality of Rotterdam, 2023) was consulted, including media materials such as videos and press releases; for Helsinki the website “Helsinki Smart” (Helsinki Smart, 2022) was consulted, to which the report on “Kalasatama Digital Twin”^1^ was added. On the other hand, the analysis benefited from a series of interviews with municipal officials and tech experts involved in the chosen case studies. Hence, primary literature, official documents, and interviews establish a dialogue in which to none is granted a privileged role; rather, it is through their dialectics that it becomes possible: (1) to obtain a first-hand deeper understanding of CDT as an evolving concept; (2) to get a cognizant overview of the state of the art of these projects; (3) to highlight opportunities and hindrances toward the development of CDTs from a sociotechnical and governance perspectives.

Six interviews were collected between September 2022 and January 2023, involving three leading representatives (one for each of the chosen case studies: one engineer with project management responsibilities in Dublin; one urban planner and engineer in Helsinki; one leading project manager in Rotterdam). Questions during the first round of interviews aimed (a) to get an overview of the ongoing CDT projects in each city, as well as the visions behind them and the approaches followed; (b) the stakeholders involved and their responsibilities; (c) the main sociotechnical challenges faced. In the second round of interviews, questions revolved around CDT as a concept and its evolution over time, notably (d) how it is different from 3D/4D/nD models; (e) how the concept has evolved over the last 5 years; (f) how it might evolve from a technical (e.g., integrated real-time digital modeling) and social perspective (e.g., as a tool for urban policy and decision-making) over the next 5 years. While diverse public and private actors do inform the development, implementation, and use of digital twin technologies in/for the city, here the focus is on municipal officials and tech experts working within/for the selected municipalities as they are public actors bound to transparency and to act in view of the collective interest, thus maintaining in principle a societal outlook next to an economical one concerning the digitalization of the urban environment.

Based on the limits of current CDTs identified in Section 3 above, the study focuses on three levels of analysis: (i) technical; (ii) sociotechnical; and (iii) governance. Concerning the technical level, the analysis explored the technical deployment of the identified CDT projects, that is, the extent to which they concretely enact an integrated, real-time modeling of the city, as per definition, or, by contrast, the extent to which they largely remain 3D static models. Hence, documents and interviews were searched for notations to technical aspects of CDTs, such as current barriers, limitations, as well as future potentialities.

Concerning the sociotechnical level, the analysis investigated if/how these projects take into account non-technical aspects, from policy to participation. Hence, documents and interviews were searched for references to a broader contextual societal dimension, including the identification of urban problems to be tackled by/through CDTs, the inscription of CDTs into a broader agenda of urban development, and the involvement of citizens into the development and use of CDT.

**Figure 1:**
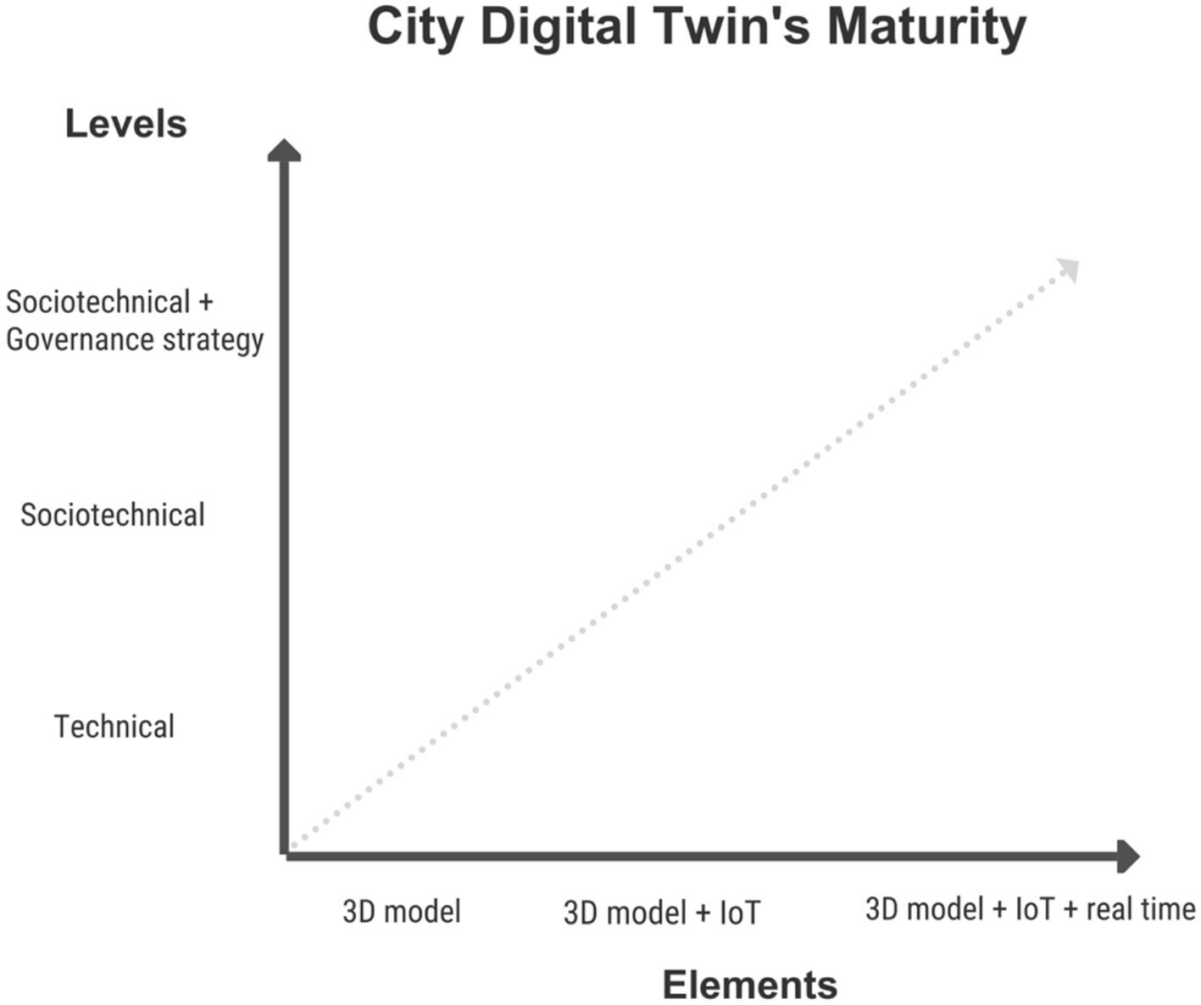
Coordinates for assessing the maturity of CDTs.

Last, the governance level was mainly concerned with assessing whether these projects are supported by strategic visions (e.g., short-term and/or long-term), with exploring the kind of governance they rely upon (e.g., which actors involved, top-down vs bottom-up approach, private- and/or public-led), as well as with understanding the extent to which CDTs are meant to support policymaking. Figure 1 identifies the coordinates of the study, visualizing the levels of analysis—technical, sociotechnical, and governance—the key aspects of a proper CDT—integration and real time synchronization of a city 3D model with diffused IoT data—and its implied trajectory of maturity.

## 6. Case studies: Dublin, Helsinki, and Rotterdam

### 6.1. Dublin

The roots of the “Smart Dublin” initiative (Dublin City Council, 2022) date back to mid 2010s, with one of the earliest milestones being the 2018 rebranding of the Docklands district as “smart.” A convergence in loco, bringing together tech enterprises, academic institutions, and public sector officials, ensued with the goal to experiment new solutions to revamp the livability and sustainability of the neighborhood. This experience was soon replicated across four other Dublin districts: DCU (the University campus), Sandyford (business district), Balbriggan (community-building), and D8 (health neighborhood). Today, the Smart Dublin initiative includes four local authorities, five smart districts, and a cohort of private partners (both tech giants such as Google and Microsoft, as well as SMEs), research centers, and public sector agencies.

In parallel to the launch of the first smart district, the Smart Dublin initiative created the premises for a digital twin of the city. In 2019, the municipality organized a data hackathon open to all, from which to pool stakeholders to design city 3D models based on open government data. As Jamie Cudden (Bee Smart City, 2021), at the head of Smart Dublin, reveals: “Before we established Smart Dublin (…) it was more like we were looking for problems to solve with technology rather than using technology to solve problems.” While being publicly led at municipal level, through the hackathon it was especially private companies to be endowed with the task to deliver models and projects, according to identified priorities. More specifically, the strategy for Dublin's CDT is based on the ongoing consolidation of five pillars: (1) Emergency Services; (2) Prediction Analytics & Real Time Data; (3) Sustainability & Climate Change; (4) Tourism & Culture; and (5) Future of Planning & Consultation. While these areas of development and application of CDT have mid- to long-term viability, at the moment the scenario remains fragmented, in terms of (1) diversity (with projects that remain siloed and led by different actors); and (2) maturity (with projects often at an early stage of development).

Concerning diversity, currently Smart Dublin has fostered an ecosystemic vision (Dublin City Council, 2022) without a proper core: its municipality-coordinated lead favors a lean approach toward the digitalization of the city that makes room for the fostering of public-private synergies on a case- by-case scenario, leaving up to each project the modalities of its deployment. In prospect, this might lead to the development of several CDTs for the same city as a more easy-to-handle modular approach to the digitalization of the city (Wan et al., 2019). And yet, there remains the issue of integrating or at least loosely coupling together (Radenkovic et al., 2020) differently designed DTs. It does not surprise that the interviewed leading engineer part of the smart city unit of Dublin city council claimed that “one of their biggest challenges is currently the interoperability across the projects developed by different stakeholders.”

Concerning maturity, Dublin case shows that the coming into being of a proper CDT is still to come. Currently, indeed, the project enacts a 3D model-centered idea with a focus on how to integrate different data coming from diverse sources (e.g., IoT) into the model. As it was specified during the interview, “a DT is not only GIS and BIM; it is all this and more. It is about connecting data layers and take evidence-based decisions.” While data integration is on the agenda, currently it remains a desideratum rather than an effective result. This “more” to which the interviewee refers, indeed, hints at the pillars of the project still in need of consolidation, notably the real-time mutual synchronization between the DT and its physical counterpart (“prediction analytics and real time data”), and the possibility to eventually use the CDT as a policy-making compass (“future planning”), including citizen involvement (“consultation”). According to the interviewee, the CDT is not yet policy-oriented tool due to a lack of cross-project coordination, while public consultation remains scant because it is expensive to launch and maintain.

At the same time, in a recent article White et al. (2021) discuss the development of a CDT-pilot of the Docklands district in Dublin based on Dublinked open data (the city's open data portal). The model is composed of six layers—terrain, building, infrastructure, mobility, digital layer (real-time data), and virtual layer (real-time information processing)—allowing for diverse urban planning simulations to be realized. This design approach follows up on Castelli et al.'s (2019) suggestion about organizing the CDT into layers to facilitate the integration among the city's various domains and dimensions. Furthermore, being openly accessible and interactive, the Docklands' CDT allows citizens not only to intuitively grasp the impact on the built environment of suggested changes, but also to immerse into the 3D model through AR and VR technologies, eventually providing feedback and making propositions. This resonates with what Dembski et al. (2020) have done in Herrenberg, Germany, where they created a CDT and adopted VR to discuss with citizens possible solutions to traffic congestion. These pilots show the potentialities of CDT for public consultation and participation in urban development-related decisions. Currently, however, in Dublin's case public consultation is not in the loop yet, but largely summoned upon concerning already identified ideas, thus enacting what Cardullo and Kitchin (2019) call “forms of stewardship and civic paternalism.” As the interviewee claimed, “citizens demand transparency… what has been done so far is to pre-identify some areas of intervention and solutions and submit these to public scrutiny (facilitated by VR).” While this does represent a valuable first step, to make civic consultation and participation cognizant and effective, there is the need to embed citizens into the whole decisional process and favor engagement in the long run (Toots, 2019).

The risk of tokened civic participation (Arnstein, 1969) emerges vividly with regard to city dashboards, concerning which Dublin is also a leading example. Building City Dashboards (BCD) was a project financed by Science Foundation Ireland, part of Smart Dublin ecosystem, and ran from 2016 to 2020 (the dashboard was accessible online until January 2022). The goal was to improve the existing Dublin (and Cork) dashboard, investigating and testing a new suite of querying, modeling, and prediction/simulation modules, as well as novel interactivity, including virtual reality (Dawkins et al., 2021). More broadly, city dashboards enact new managerialist approaches to the public sector, which promote real-time monitoring of cities through openly accessible and re-usable datasets.

On the one hand, dashboards do represent “cognitive tools” (Dawkins et al., 2021) to make sense of big data, especially for unskilled users who might not be able to extrapolate significant correlations and trends from available datasets. As such, dashboards can contribute to shape a more informed citizenry about urban issues, and one that is willing to participate in decision-making processes about the city's development. On the other hand, as Dawkins et al. (2021) note, most of the time “knowledge and understanding of user requirements is assumed without meaningful engagement of potential users in the process of scoping and design.” This means that dashboards are conceptualized, designed, and implemented without a sufficiently robust understanding of the diverse targets they might reach and/or their needs not only as users, but citizens.

Disenfranchised from a proper context-based assessment, the danger with dashboards is to see complex governmental practices metamorphosed into products to “play with” and to eventually evaluate, rather than as a collectively informed decision-making process. In other words, city dashboarding risks coopting citizens instead of concretely involving them. Literature (Toots, 2019) shows that it is especially when citizens are dutifully informed and endowed with deliberative power that they are most willing to participate. It is crucial, then, not only to focus on how to overcome issues of “access, quality, comprehensibility and limited scope of data” (Dawkins et al., 2021) upon which city dashboards are built, but, more broadly, to manage the digitalization of the urban environment as “a process of continuous innovation, learning and adaptation” (Toots, 2019). Put differently, city dashboards shall be regarded as part of a broader sociotechnical dimension (1) supported by long-term vision and planning and (2) allowing all stakeholders to get involved in the whole decision-making process. These two aspects are deeply intertwined. The Smart Dublin initiative does show a long-term vision and growth, as the pillars discussed above indicate. Yet, according to the interviewee, currently this vision and growth are still at a preliminary stage and largely relying on private companies with consequent interoperable challenges and open questions on the viability of CDTs as compass for policymaking and non-tokenized consultation.

### 6.2. Helsinki

Helsinki is home of one of the longest CDT initiatives in Europe (Hämäläinen, 2020). Earliest 3D models of the city date back to 1987, while the idea to capitalize on these models to create a digital twin of the city originated in the early 2000s. Today, Helsinki is part of a regional innovation ecosystem comprising large businesses and start-ups, public sector, research and education centers (Helsinki Smart, 2022). A city information model for the entire capital was created in 2017 and is being periodically updated by the Helsinki City Survey Services in the City Urban Environment Division.

The first district to undergo a digital twinning process in Helsinki was Kalasatama, a small waterfront area in the east part of the capital. The Kalasatama CDT uses open government data to deliver a high-quality 3D model of the district (using CityGML over five levels of details), including current, under-construction, and future buildings, as well as bridges, water areas, and terrain. In this respect, the city information model is one of the most detailed in Europe. According to the official report (see text footnote^1^), the overall objectives of the CDT project are (1) to establish an open-access testbed for monitoring the lifecycle of the built environment; (2) to develop an open-source platform where the digital twin can provide the basis for interaction with residents; (3) to embed the CDT into broader decision-making processes concerning urban development and the delivery of services to citizens and visitors.

Concerning point 1, currently the open access 3D model guarantees free-of-charge accessibility to anyone. The project is municipality-centric and largely public-led, with “private actors mostly as suppliers of applications,” as the strategy department official pointed out in the first interview. Particular attention is also paid to the usability of the model to favor an independent usage by end users, communities, as well as for educational purposes.^2^ On this point, the interviewee mentioned that “being based on lots of open data, we can aggregate different data sources to deliver intuitive analyses on urban performances, but it's mostly visualization.” In this respect, while to build the CDT upon open government data can enable transparency and facilitate public scrutiny and engagement, visualization affordances might not suffice, *per se*, to entice such quest for transparency, scrutiny and especially engagement. Open government data initiatives have shown limitations especially with regards to data's fine-tuning with the needs of local end users. Data supply alone does not lead straightforwardly to the creation of public value: as Welle Donker and van Loenen (2017) stress, it is important to be in touch with societal issues, while matching demand and supply of data. Clearly, at stake is a matter of knowing which data are needed, by whom, and for which purposes. On this point, Lupi et al. (2002) call for the delivery of “appropriate data” as the baseline to make sure that the whole smartening of cities follows and responds to indigenous dynamics. In other words, efforts into the democratization of urban data, as a synergy between top-down and bottom-up stances, remains essential to secure an inclusive modeling of CDTs. This is also why scholars have called to action to mobilize authorities at various levels for engaging citizens and foster stakeholder communities around open data (Mergel et al., 2018).

Concerning point 2, Smart Kalasatama was established by the city council as the flagship project for the smartening of Helsinki (see text footnote^1^). Smart Kalasatama revolves around the initiatives of Kalasatama Urban Lab, attracting multiple stakeholders including businesses, public actors, and residents. To consolidate this approach, a CDT platform was created based on the browse-based Open City Planner, which was subsequently acquired by the private company Bentley Systems, leading to a potential interoperability issue to be harmonized. Notably, speaking of the technique of ground control points' measurement as the basic step to enable an accurate 3D model, the report (see text footnote^1^) write that this technique was done relying on MicroStation software, owned by Bentley Systems; moreover, “at the end of 2018, Bentley Systems acquired Agency9, which originally created the OpenCities Planner application. MicroStation is a product owned by Bentley Systems, so in future interoperability between MicroStation and OpenCities Planner is expected to develop.”

Notwithstanding this issue, the platform enables interactivity and supports the use of queries and feedback: for instance, on one occasion residents were asked to indicate which Kalasatama's locations they would recommend to tourists (Hämäläinen, 2021). Yet, while promoting civic consultation, the platform does not really enable an organic participation process with deliberative power. This aligns with the study by Charitonidou (2022), according to which “despite the fact that often the dissemination of urban scale digital twins focuses on their aspiration to enhance the participation of citizens in the decision-making processes, this is not valid in most of the cases.” In the case of Helsinki, the choice of allowing limited-by-design participation has economic and governance motivations. As the interviewee claimed during the first interview: “we know what the needs are already; people need more education to understand the capabilities of the model; but there is not enough funding to do that.” This signals not only the will of the municipality to keep a top-down control over the unfolding of the CDT, but also the limited institutionalization of the initiative into municipal budgeting flows, which currently circumscribe CDT projects to small scale pilots not backed by citizenry's data literacy enhancement.

In terms of sector priorities, currently the municipality aims to develop accurate CDTs to narrow down on issues of mobility and buildings' energy efficiency: “we have small projects going on… We are not even aiming to develop one digital twin, but to foster interoperability across different projects.” This aligns with exploratory research conducted by Hämäläinen (2021), whereby “current 3D data modeling and digital twin technologies serve the city best when the aim is to model a specific city function or use case.” Overall, while Helsinki is doing well in integrating multiple sources of data in each pilot, interoperability across projects and real-time optimization remain open concerns. As the interviewee argued in the second interview, “currently, there is no coordination yet across projects… the plan is to provide more frequently updated information cycles… but 3D-models may or may not be a part of the data used… I think city digital twin work will continue with more focus on data management, data governance and advanced analysis tools, with less focus on 3D-models.”

The quote above links more closely to point 3, that is, the plan to make the CDT a tool to enable broader decision-making processes. As it is stated in the report, “the project is carried out in stages, and the final objective is to achieve operations that are holistically based on information models. This requires new kinds of expertise, adequate resources and a roadmap to guide changes.” This goes in the direction of a process-based understanding of CDT that extends beyond its technical operability, identifying non-technical conditions—from tech-legal capacities to data literacies and organizational culture—to enable a sociotechnical enactment of the twinning. Yet, 6 years into the publication of the report, technical issues continue to play a major role, as claimed by the interviews, preventing an orchestrated governance-based implementation of CDTs and an effective enactment of these as policy-oriented tools; similarly, while citizens have been summoned upon in some projects, a full-fledged sociotechnical approach implying a systemic involvement of citizens remains out of reach.

### 6.3. Rotterdam

Initial steps to design a CDT for the city of Rotterdam—a “Digitale Stad” (trans. “Digital City”)—started at the end of 2018, although “we were already working on the idea of DT before this existed, so we used this [term] to give a name to what we were already working on,” as the program manager of the municipality pointed out. For one thing, this is one further confirmation of the extent to which DT as a concept has entered agendas of European municipalities, also shaping discourses of urban technological innovation around it.

Rotterdam's CDT follows an integrated approach bringing together three interlaced dimensions: physical, digital, and societal (Urban Big Data, 2022). Cutting across these three dimensions, the CDT has the goal to provide an all-encompassing real-time replica of the entire city. Since the outset, then, the municipality of Rotterdam, which leads and controls the implementation of the project, recognized the need to calibrate the digital modeling of the city in a dynamic way (i.e., real-time) and incorporating not only infrastructural performances, but also non-physical fluxes such as social and economic processes: “for us,” the program manager noted in the second interview, “the CDT is a GIS-informed nD model, combined with real-time data that describes the functioning of the city.” In this regard, the team in charge of Rotterdam's CDT has taken a holistic approach by default and opted for the development of one platform-based centralized DT, accessible and usable by a variety of stakeholders, according to different needs. As the project manager noted, the CDT will enable the delivery of “all kinds of smart solutions and services, including city dashboards, decision-making, participation/co-creation, permit-processes, physical safety, sustainability.” Currently, 4 years since its formal inception, the project has entered the implementation phase.

Overall, the municipality has devised two working groups around its CDT: one dedicated to enabling data interoperability and the delivery of technical support, the other focused on exploring non-technical issues. As of January 2023, the technical team has finalized the development of the Open Urban Platform (OUP): “the next step is to connect it to the social reality and create a Digital Urban Community (DUC). This DUC touches upon everything that has to do with the metaverse” as well as with “training capabilities of citizens and civil servants.” Notably, it is on and through the OUP that technical and non-technical aspects are expected to join and inform each other: “when we were thinking about the OUP,” the program manager recalled, “we had in mind not ourselves [municipality], but the whole city: ‘How to design the right platform?' And yet, it was not only a matter of design: a successful ecosystem is based on trust among all parties.”

At this stage, the interdependence among technical and non-technical aspects (and actors) has not become effective yet: currently, the Zwolle-based company Future Insight partnered with Capgemini and the municipality of Rotterdam to develop the OUP as a web-based infrastructure (the agreement is for 10 years). As it is possible to read on the website of the initiative (Municipality of Rotterdam, 2023), the platform is fully developed with flexible technical components (MIMs) that are connected to each other by means of established open interfaces. This is meant to favor flexibility and scalability, minimizing lock-in risks. Importantly, to make sure that the platform complies with ethical standards and privacy-sensitive matters, an independent governance board will be set up, with the goal to monitor the operation of/on the OUP.

Technically speaking, the platform will represent the infrastructure for managing data coming from multiple sources and implementing applications and services on top of that. According to the initiative's website, these applications and services refer to both themes (e.g., sustainability, safety, etc.) and processes (e.g., co-creation, planning, etc.). It is especially processes that demand a more orchestrated effort in terms of design and multistakeholder collaboration before achieving effective operationalization. The intention is to have the OUP to function as a publicly monitored catalyst around which to pool private actors, academia, as well as citizens in the fostering of digital services that benefit the city as a whole. Similar to a dashboard, the platform will represent the visually intuitive, navigable and interactive interface of the data lake; yet, more radically than a dashboard, the platform will be expected to constitute a “joint data foundation” (Municipality of Rotterdam, 2023). At present, however, it is uncertain how this foundation will be concretely designed and realized. From a governance perspective, if, on the one hand, this could lead to a data federated model, for instance in the form of a public data trust (Micheli et al., 2020); on the other end, to have the whole CDT gravitating around the OUP might produce forms of platformization (van Dijck et al., 2019) and power asymmetries within the data ecosystem.

On the website of the Municipality of Rotterdam (2023), it is stated that “the CDT will not only enhance participation but allow for co-creation. There are several sharing and preview options, such as QR, Virtual Reality and Augmented Reality.” In fact, the OUP is expected to be informed by and meant for different user groups—Municipality, Knowledge Institutions, Companies SMEs, Housing Corporations, Energy Suppliers, Municipalities, Residents and Visitors—which will both supply data and use data. This seems to suggest the enactment on and the through the OUP of a governance model that does strive for multistakeholder inclusiveness and co-creation having in mind the city as a heterogeneous sociotechnical dimension. However, the extent to which such enactment will avoid cooptation will greatly depend on the fostering of a robust and cognizant DUP, as well as of trustworthy mechanisms to prevent the centralization of data power, for which the independent governance board will play a crucial role. Given the current early phase of the whole CDT initiative—de facto Rotterdam is the youngest of the three reviewed—these points all warrant further exploration in the upcoming years.

## 7. Discussion

Data for this study was primarily collected by interviewing CDT officials and tech experts involved in the three case studies, as well as by incorporating gray literature—e.g., reports and official websites—with relevant primary literature, e.g., on participation, dashboards, platformization.

From a sociotechnical and governance perspectives, a comparison among the three case studies reveals significant trends concerning different paths (and stages) for the conception, development and implementation of CDTs (Table 1). To begin with, while being all longstanding initiatives, all three CDTs are still at an early stage of development, aligning these findings to recent review (Ferré-Bigorra et al., 2022).

**Table 1:** A summary of technical, sociotechnical and governance aspects of city digital twins in Dublin, Helsinki and Rotterdam.

**Aspects & cities**		**Dublin**	**Helsinki**	**Rotterdam**
Technical	Model	BIM + GIS + (limited) IoT	(OGD-based) CIM + BIM + GIS	BIM + GIS + (limited) IoT
	Integration	Limited (multiple CDTs)	Limited (multiple CDTs)	n.a. (expected 1 CDT)
	Real-time	(OGD-based) pilots	Pilots	n.a. (expected 1 CDT)
Sociotechnical	Policy compass	Very limited	Very limited	n.a.
	Particiaption	Consultation, feedback, navigation	Navigation, feedback	(Expected) consultation, co-creation
Governance	Vision	Cyberphysical ongoing knowledge across areas and sectors	Integrated sychronicity & sectorial CDTs	Merge of physical, digital, and social dimensions
	Model	Multistakeholders, lean approach, municipality-led, outsourced implementation	Municipality-centric, top-down, private & public actors as suppliers	Multistakeholders, municipality-led, paltform centric (OUP + DUP)

Sociotechnically speaking, this means that, beyond optimistic long-term claims, the use of CDTs as policy-orienting tools and/or their use for active civic engagement, currently remain more *desiderata* than concrete achievements. The pandemic might have slowed the CDTs' development and implementation, especially due to already limited or shrinking funding; however, the analysis also highlighted through the interviews that it is especially the difficulty to realize the digital twinning of a complex system—as cities are—to be challenging. As it happened in the past with smart solutions, a practice-first tech-centered approach risks delivering (the illusion of) short-term economic-efficient fixies, while the implementation of a multilayered infrastructure-based and service-oriented digitalization of the urban environment demands an orchestrated approach informing and informed by non-technical factors that must be taken into account upfront rather than considered along the way. Notably, the study highlighted that the epistemological value of CDTs currently remains confined to experts, while citizens are summoned upon only for consultation or feedback at later stages. This, in the long run, might risk reinforcing or perpetrating a siloed approach to urban development, without guaranteeing sufficient agency to local communities.

These limitations also reflect the governance behind the explored case studies. If, on the one hand, all three cases espouse a multistakeholder governance modulated, as envisioned by exiting literature, on the quadruple helix—municipalities, private actors, academia, and citizens—it is the distribution of power within each model to be different. Dublin favors a lean approach that allocates great power and technological discretion to partners—mostly private companies—a aligning more closely to “traditional” smart city agendas as discussed in literature; Helsinki adopts a municipality-centric approach that in principle grants accountability and transparency, but struggles to exit its institutionalized lock-in with the consequence of (more or less intentionally) limiting the reaching out toward citizenry; Rotterdam, albeit having two working groups within the municipality that are focused on technical and non-technical issues, opts for having the convergence of stakeholders as both data users and suppliers on a single platform with potential effects on positive synergies as well as risks of power asymmetries.

From a technical point of view, although all three cases point, in the long run, to a cyber-physical holistic development of CDTs, currently these projects remain anchored to city information models synthetizing GIS and BIM with some IoT-derived data. Hence, integration and interoperability remain major issues to be tackled, as evidenced also by recent literature (Lei et al., 2023; Weil et al., 2023). In fact, in Dublin and Helsinki it remains to see if/how the coordination across various projects, as a result of their decision to develop multiple CDTs, will be tackled. This is especially relevant in the case of Dublin, which tends to outsource projects mainly to private stakeholders. Real-timeness, in turn, is still to come in that this key aspect of a proper DT currently faces technical barriers as well as limited organizational capacities (usually the teams behind these projects are quite small). Rotterdam is the case that prioritizes the most an integrated and all-encompassing approach by default for the design of its CDT; yet it is also the least developed project so far and its concrete implementation requires further exploration. In fact, Rotterdam's choice (and effects) to go for the design of one city-wide DT warrants an assessment in the long run, in terms of its accessibility, usability, and management, especially the launch and maintenance of a digital urban community and an independent governance board.

**Figure 2:**
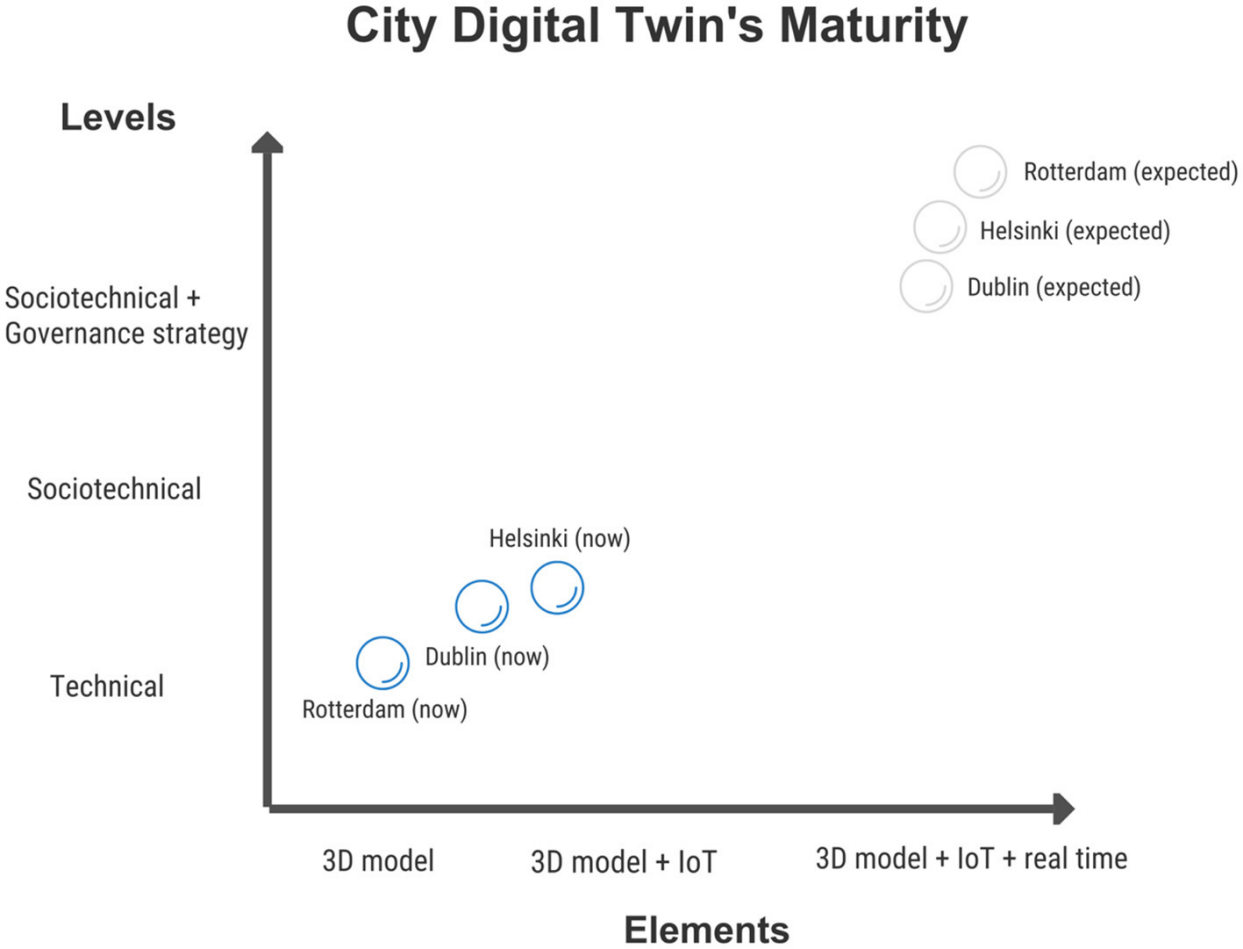
Current and envisioned CDTs' maturity of Dublin, Helsinki and Rotterdam.

Figure 2 updates the infographic presented in Section 4, visualizing the actual maturity of these projects (darker dots) and as these are expected to evolve in the years to come (within 6–8 years; lighter dots). Currently, Helsinki is the most advanced CDT, especially from a technical perspective, while all three cases show scant evidence when it comes to unlock the enablers to a full-fledged sociotechnical approach and orchestrated governance across projects (interoperability) and actors (including citizens). Differences in expectations among the three cases are very limited; yet, Rotterdam will foster since the outset an approach aimed at merging top-down and bottom-up stances, Helsinki will maintain a municipality-centric approach throughout, while Dublin will follow up on a lean approach that will guarantee adaptation to a case-by-case scenario.

## 8. Conclusion

The analysis showed that while the concept of DT has made it into European municipalities' agendas (especially of big cities), its concrete implementation is still in its infancy, concerning both its proper development as a fully integrated bi-directional (physical-digital) city modeling, as well as a broader sociotechnical framing of the digital twinning of the urban environment and an orchestrated governance across projects and actors.

If Gartner cycled preconized the mainstream adoption of DT by 2027, in the urban context this will likely take longer. This—for once—might not be bad news, insofar as a slower development might favor and reflect the need for a cognizant integration of technical and non-technical factors, as the Rotterdam case seems to suggest. Overall, while technical barriers will be likely overcome thanks to ongoing innovation, especially in data semantics and data fusion and through increased computer processing capacity, other non-technical barriers, such as limited tech expertise in public sector, limited data literacies in citizens, as well as the fostering of an organization culture prone to change, demand orchestrated strategies. The sooner these will be devised, and put into action, the better, since their impact will likely be visible only in the mid to long term. At the same time, the analysis shed some light onto the potentials of what CDTs can do, how, and for whom, with 3D modeling used to tackle mobility issues, monitor buildings' energy consumptions, as well as asking feedback to citizens on urban related issues. Yet, for these uses to be fully integrated into participated urban development plans CDTs need to avoid reification and be rethought and redesigned as ongoing processes.

It is a whole new urban sociotechnical dimension that is emerging, and this requires case- study latitudinal studies, as well as longitudinal ones. In one respect, future research might fruitfully look into how cities of different sizes and/or beyond the EU pursue their goal of achieving a digital twinning of the urban environment; in another respect, research might look back, in a few years, at the case studies presented here to explore the stage of development of their CDTs and draw valuable lessons for other cities following up on their wave. Another option is to widen and diversify the cohort of stakeholders beyond the city officials and tech experts interviewed here, acquiring valuable insights from private actors as well as citizens. Alternatively, it would be worth turning things upside down and exploring (the existence of) cases that foreground the consolidation of robust sociotechnical bases—i.e., data literacies and co-creative urban development processes—as *preliminary conditions* (and not only optional *desiderata*) to any smart city agenda.

## Data availability statement

The original contributions presented in the study are included in the article/supplementary material, further inquiries can be directed to the corresponding author.

## Author contributions

The author confirms being the sole contributor of this work and has approved it for publication.

## Footnotes

^1^ http://www.hel.fi/static/liitteet-2019/Kaupunginkanslia/Helsinki3D_Kalasatama_Digital_Twins.pdf

^2^In 2018, for a period of 6 months, the model was also accessible in streaming, as a visually interactive and navigable.
